# The "smoker's paradox" in patients with acute coronary syndrome: a systematic review

**DOI:** 10.1186/1741-7015-9-97

**Published:** 2011-08-23

**Authors:** Erlend Aune, Jo Røislien, Mariann Mathisen, Dag S Thelle, Jan Erik Otterstad

**Affiliations:** 1 Department of Cardiology, Vestfold Hospital Trust, Tønsberg, Norway; 2 Department of Biostatistics, Institute of Basic Medical Sciences, University of Oslo, Oslo, Norway; 3 Morbid Obesity Centre, Vestfold Hospital Trust, Tønsberg, Norway; 4 Medical Library, Vestfold Hospital Trust, Tønsberg, Norway

## Abstract

**Background:**

Smokers have been shown to have lower mortality after acute coronary syndrome than non-smokers. This has been attributed to the younger age, lower co-morbidity, more aggressive treatment and lower risk profile of the smoker. Some studies, however, have used multivariate analyses to show a residual survival benefit for smokers; that is, the "smoker's paradox". The aim of this study was, therefore, to perform a systematic review of the literature and evidence surrounding the existence of the "smoker's paradox".

**Methods:**

Relevant studies published by September 2010 were identified through literature searches using EMBASE (from 1980), MEDLINE (from 1963) and the Cochrane Central Register of Controlled Trials, with a combination of text words and subject headings used. English-language original articles were included if they presented data on hospitalised patients with defined acute coronary syndrome, reported at least in-hospital mortality, had a clear definition of smoking status (including ex-smokers), presented crude and adjusted mortality data with effect estimates, and had a study sample of > 100 smokers and > 100 non-smokers. Two investigators independently reviewed all titles and abstracts in order to identify potentially relevant articles, with any discrepancies resolved by repeated review and discussion.

**Results:**

A total of 978 citations were identified, with 18 citations from 17 studies included thereafter. Six studies (one observational study, three registries and two randomised controlled trials on thrombolytic treatment) observed a "smoker's paradox". Between the 1980s and 1990s these studies enrolled patients with acute myocardial infarction (AMI) according to criteria similar to the World Health Organisation criteria from 1979. Among the remaining 11 studies not supporting the existence of the paradox, five studies represented patients undergoing contemporary management.

**Conclusion:**

The "smoker's paradox" was observed in some studies of AMI patients in the pre-thrombolytic and thrombolytic era, whereas no studies of a contemporary population with acute coronary syndrome have found evidence for such a paradox.

## Background

The term "smoker's paradox" was introduced into scientific discourse more than 25 years ago following observations that smokers (in comparison to non-smokers) experience decreased mortality following an acute myocardial infarction (AMI) [1-4]. Braunwald's recent textbook on heart disease argues that the observation that smoking predicts better outcome following various reperfusion strategies is not because of any benefit from smoking but simply because smokers are likely to undergo such procedures at a much younger age and hence have on average lower comorbidity [5].

In a recent study we observed a 41% reduction in one-year mortality in unselected AMI patients following the switch from a conservative approach in 2003 to the introduction of routine early invasive management in 2006 [6]. In a sub-analysis of patients with non-ST-segment elevation myocardial infarction (NSTEMI) this treatment effect was especially pronounced for smokers. Current smoking was an independent predictor for one-year mortality in the 2003 cohort, but not in the 2006 cohort [7]. These observations motivated us to perform a systematic review of the literature (observational studies and randomised trials) surrounding the "smoker's paradox" in order to explore possible differences between study populations with or without this phenomenon.

## Methods

### Literature search and study selection

We searched three electronic databases: EMBASE (from 1980 onward), MEDLINE (from 1963 onward) and the Cochrane Register of Controlled Trials. Our search strategy combined text words and subject headings identifying reports relating to acute coronary syndrome/AMI, smoking status and mortality. The search included literature published by 22 September 2010. Due to the long time spans of the databases we decided to perform two slightly different searches in MEDLINE and EMBASE, one from 1963/1980 to 1995, the other from 1996 to date of search. (See Additional file 1 for the full search strategy.) The reference lists of identified studies were also scanned to identify any other relevant studies, with the search strategy expanding accordingly.

The original observations of the "smoker's paradox" was made in patients with an AMI diagnosed according to the World Health Organisation (WHO) criteria from 1979 [8]. With the introduction for new diagnostic criteria of AMI in 2000 [9] and 2007 [10] in mind, we chose to extend the search to include patients with ST-segment elevation myocardial infarction (STEMI) and non-ST-segment elevation acute coronary syndrome (NSTEMI and unstable angina pectoris [UAP]).

Two investigators (EA and JEO) independently reviewed all titles and abstracts to identify potentially relevant articles and resolved discrepancies by repeated review and discussion. If two or more studies presented the same data from a single patient population, we included these data only once in the review.

No review protocol was used, but we prospectively defined the following criteria for the inclusion of studies into our review:

• Studies of patients hospitalised for acute coronary syndrome (ACS), including the previous WHO criteria for AMI [8] and the more recent definition of ACS, including STEMI, NSTEMI and UAP [9].

• The publication should provide a clear definition of smoking status into current, former and never-smokers, including baseline characteristics of each group with age as a minimum. In case former smokers were not defined separately, a minimum requisite was that they had to be defined and characterised either as smokers, non-smokers or per definition were excluded from the analysis.

• Both crude and adjusted total mortality rates should be presented. Effect estimates should be provided, and "age" was a minimum requirement as a covariate.

• The length of follow-up should be reported and include at least hospital mortality. Studies reporting only post-discharge mortality were excluded.

• Only English-language original articles were included.

• The study should include > 100 smokers and > 100 non-smokers.

Our own study exploring one-year mortality among smokers vs. non-smokers with NSTEMI was published after the literature search was finalised, but the results were known to us by September 2010, and the study has therefore been included in this review [7].

## Results

**Figure 1:**
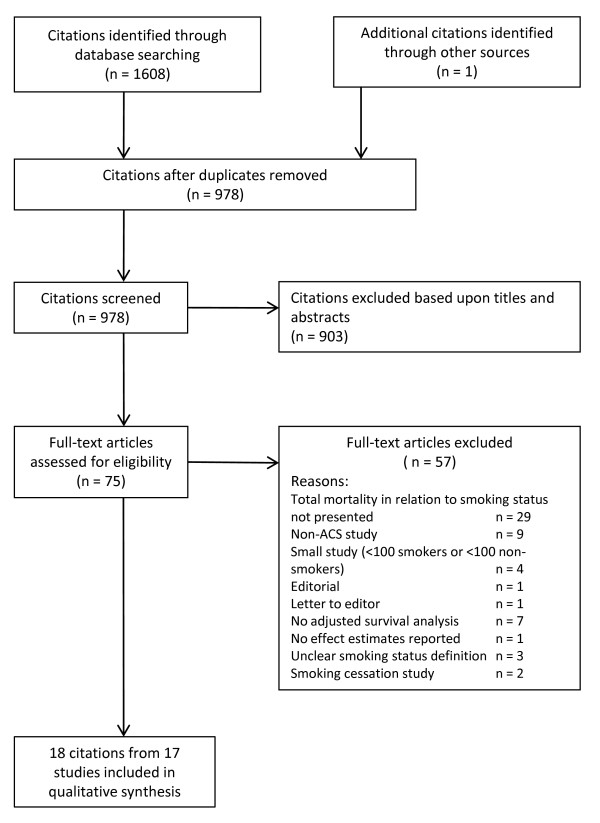
**Selection of studies**.

The study selection process is presented in Figure 1. In total, 978 unique citations were identified. Based upon titles and abstracts, 903 citations could be excluded. Accordingly, 75 full-length original articles were considered in depth for inclusion, with 18 publications from 17 studies (7 randomised trials and 10 observational studies/registries) meeting all inclusion criteria [7,11-27]. The Superior Yield of the New strategy of Enoxaparin, Revascularization and GlYcoprotein IIb/IIIa inhibitors (SYNERGY) trial is presented by two publications, one demonstrating crude mortality rates [22] and another adjusted mortality rates [24]. The studies were published between 1991 and 2009 and enrolled patients from 1982 through till 2007. Five studies [7,14,16,19,22,24] were considered to represent a contemporary population of ACS and mainly included patients according to the diagnostic criteria from 2000 [9]. The other studies were based upon patients included according to the WHO criteria [8] in the 1980s and 1990s.

The follow-up time in the 17 included studies varied from in-hospital to three years. Out of six studies with in-hospital follow-up, two registries demonstrated a "smoker's paradox". Four out of six studies with follow-up between one month and six months found evidence for the paradox, whereas none of the five studies that followed patients for one year or more did so.

### Study categories and adjusted mortality rates

**Figure 2:**
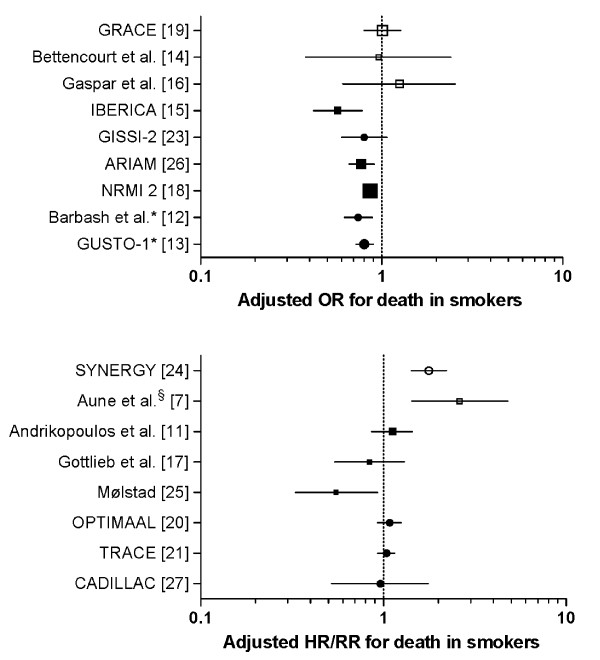
**Forest plots of adjusted mortality in smokers vs. non-smokers**. Odds ratios (OR)/hazard ratios (HR) with 95% confidence intervals for death during follow-up for smokers compared with non-smokers in the studies included. Circles indicate data derived from randomised trials. Squares indicate data derived from observational studies or registries. Open symbols indicate contemporary studies enrolling patients mainly after 2000. Closed symbols indicate older studies enrolling patients in the pre-thrombolytic and thrombolytic era. Symbol size reflects the sample size of the studies and registries. *Inverted OR from original paper. ^§^The adjusted HR is for the conservative treatment cohort (2003) only. For the invasive cohort (2006) there was no difference in mortality for smokers and non-smokers (data not published).

Study characteristics, with crude and adjusted mortality rates expressed as odds ratios and hazard ratios and relative risks with 95% confidence intervals according to smoking status, are presented in Table 1. The studies have been sub-divided into six categories according to study design. The effect estimates for adjusted mortality rates are presented in Figure 2.

**Table 1:** Study characteristics and mortality rates according to smoking status at index event

Study	Paradox?	Time symptoms to inclusion	**Publ**.	**Enroll**.	Index Event	n	Current (C) n (age)	Former (F) n (age)	Never (N) n (age)	Follow -up	Total mortality			Adjusted mortality rates with 95% confidence interval
												
											C	F	N	
**Randomised clinical trials in STEMI patients (thrombolytic treatment)**														
**GUSTO-1 **[13]	Yes	3.2 ± 1.65 h	1995	90 to 93	STEMI	40,599	17,507 (55)	11,117 (64)	11,975 (66)	30d	4.0%	6.7%	10.3%	OR 1.25 (1.11 to 1.39) N vs. C
**Barbash *et al***. [12]	Yes	3.0 ± 1.6 h	1993	88 to 89	STEMI	8,259	3,649 (58)	2,244 (64)	2,366 (67)	6 m	7.7%	12.1%	17.6%	OR 1.35 (1.12 to 1.61) N vs. C+F
**GISSI-2 **[23]	No	< 3 h in 70%	1998	88 to 89	STEMI	9,694	5,151 (57)	1,932 (64)	2,611 (68)	In-hosp.	4.7%	7.6%	13.8%	OR 0.80 (0.60 to 1.07) C vs. N OR 0.97 (0.70 to 1.35) F vs. N
**Randomised clinical trials in STEMI patients (invasive treatment)**														
**CADILLAC **[27]	No	< 12 h	2004	97 to 99	STEMI	2,082	898 (53)	546 (64)	638 (65)	1 y	2.9%	3.7%	6.6%	HR 0.96 (0.52 to 1.76) C vs. N
**Randomised clinical trials in patients with NSTE-ACS (invasive treatment)**														
**SYNERGY **[22,24]	No	< 24 h	2008	01 to 02	NSTE -ACS	9,971	2,404 (61)	3,491 (69)	4,076 (70)	1 y	6.5%	9.1%	6.7%	HR 1.77 (1.42 to 2.21) C vs. N
**Multi-centre post-AMI randomised trials**														
**TRACE **[21]	No	2 to 6 d	1999	90 to 92	AMI	6,485	3,341 (64)	1,420 (71)	1,724 (74)	3 y	26 to 27%	38 to 39%	42 to 43%	HR 1.04 (0.93 to 1.15) C vs. N
**OPTIMAAL **[20]	No	< 10 d	2004	98 to 99	AMI	5,475	1,832 (62)	1,867 (69)	1,776 (71)	2.7 y	16.3%	Incl. in C	19.3%	HR 1.08 (0.93 to 1.25) C+F vs. N
**Single-centre observational studies of patients with AMI**														
**Mølstad **[25]	Yes	NA	1991	82 to 84	AMI	484	184 (61)	Incl. in N	456 (70)	3 m	11 to 13%	Incl. in N	32 to 34%	HR 0.62 (0.36 to 1.04) C vs. N+F HR 0.55 (0.33 to 0.93) C vs. N+F
**Bettencourt *et al***. [14]	No	NA	2004	01 to 02	ACS	901	369 (58)	Incl. in C	532 (69)	In-hosp.	2.6%	Incl. in F	6.6%	OR 0.96 (0.38 to 2.41) C+F vs. N
**Gaspar *et al***. [16]	No	NA	2009	04 to 07	ACS	1,228	450 (58)	Incl. in C	778 (68)	6 m	9.3%	Incl. in C	13.1%	OR 1.25 (0.61 to 2.54) C+F vs. N
**Aune *et al*.^§ ^**[7]	No	NA	2010	03 to 07	NSTEMI	381	103 (63)	Incl. in N	278 (80)	1y	22%	Incl. in N	27%	HR 2.61 (1.43 to 4.79) C vs. N+F
**Registries**														
**Gottlieb *et al***. [17]	No	NA	1996	94	AMI	999	367 (57)	Incl. in N	632 (67)	6 m	7.9%	Incl. in N	21.5%	HR 0.84 (0.54 to 1.30) C vs. N+F
**Andrikopoulos *et al***. [11]	No	< 24 h	2001	93 to 94	AMI	5507	3,853 (59)	Excluded	1,654 (70)	In-hosp.	7.4%	NA	14.5%	RR 1.12 (0.86 to 1.44) C vs. N
**NRMI 2 **[18]	Yes	NA	2002	94 to 97	AMI	297,458	72,585 (58)	Incl. in N	224,871 (72)	In-hosp.	8.0%	Incl. in N	16.4%	OR 0.86 (0.83 to 0.90) C vs. N+F
**ARIAM **[26]	Yes	< 24 h criterion	2004	95 to 01	AMI	17,761	5,796 (57)	3,494 (67)	8,471 (70)	ICU/CCU	5.0%	9.3%	13.3%	OR 0.77 (0.66 to 0.91) C vs. N
					UAP	7,795	1,721	1,950	4,124	ICU/CCU	0.7%	1.0%	1.5%	OR 0.81 (0.48 to 1.36) C vs. N
**IBERICA **[15]	Yes	< 12 h in 82%	2007	97 to 98	AMI	7,796	3,057 (56)	1,730	2,839 (65)	28 d	8.9%	16.9%	20.1%	OR 0.57 (0.42 to 0.78) C vs. N
**GRACE **[19]	No	NA	2005	99 to 02	ACS	19,325	5,276 (57)	5,691 (67)	8,358 (71)	In-hosp.	3.3%	4.5%	6.9%	OR 1.01 (0.80 to 1.27) C vs. N

ACS, acute coronary syndrome; AMI, acute myocardial infarction; CCU, coronary care unit; HR, hazard ratio; ICU, intensive care unit; OR, odds ratio; NA, not available; NST-ACS, non-ST-segment acute coronary syndrome; NSTEMI, non-ST-segment elevation myocardial infarction; RR, relative risk; STEMI, ST-segment elevation myocardial infarction; UAP, unstable angina pectoris. ^§^The adjusted HR is for the conservative treatment cohort (2003) only. For the invasive cohort (2006) there was no difference in mortality for smokers and non-smokers (data not published).

#### Randomised controlled trials (RCT) in patients treated with fibrinolysis for STEMI

Both the International Tissue Plasminogen Activator/Streptokinase Mortality Trial [12] and the Global Utilization of Streptokinase and Tissue-Plasminogen Activator for Occluded Coronary Arteries (GUSTO-I) trial [13] demonstrated higher adjusted mortality rates among non-smokers, that is, supporting a smoker's paradox. For the latter study, no such effect was observed in the angiographic substudy of 2,437 patients. The Gruppo Italiano per lo Studio della Sopravvivenza nell'Infarcto Micardico (GISSI-2) trial [23] included patients with the same factorial study design as the international study [12], but did not demonstrate any reduced adjusted in-hospital mortality for smokers compared with never-smokers.

#### RCT in STEMI treated with percutaneous coronary intervention (PCI)

In the Controlled Abciximab and Device Investigation to Lower Late Angioplasty Complications (CADILLAC) trial 2,082 patients with STEMI undergoing primary PCI were randomised to either angioplasty or stenting with or without abciximab [27]. Although current smokers had a lower crude mortality rate, the adjusted analysis did not find a lower mortality than that of non-smokers.

#### RCT of non-ST-segment elevation acute coronary syndrome (NSTE-ACS) subjected to invasive management

In the SYNERGY trial [28] patients with NSTE-ACS were randomised to enoxaparin or unfractionated heparin and then underwent coronary angiography and subsequent PCI or coronary artery bypass grafting (CABG). The crude mortality rate after one year was similar among smokers and non-smokers [24]. In the adjusted analysis there was a significant mortality excess among smokers vs. non-smokers, supporting the unfavourable effect of current smoking at baseline [22].

#### Multi-centre post-AMI studies from RCTs

The TRAndolapril Cardiac Evaluation (TRACE) study consisted of 2,606 patients and aimed to determine whether patients with left ventricular dysfunction post AMI would benefit from long-term treatment with trandolapril vs. placebo [29]. In a study of 6,676 consecutive AMI patients screened for participation in the TRACE study, the long-term mortality was far lower among smokers than either ex- or non-smokers. In spite of this, the adjusted analysis did not give any evidence to support the existence of a smoker's paradox in this population [21].

The Optimal Trial In Myocardial Infarction with the Angiotensin Antagonist Losartan (OPTIMAAL) study included selected patients with AMI and evidence of heart failure for randomised treatment with captopril vs. losartan [20]. The unadjusted mortality rate among current smokers was 17% lower than among non-smokers, but this decreased risk was eliminated after adjustment for age and other baseline differences.

#### Single centre observational studies of patients with AMI

Mølstad included 484 unselected AMI patients between 1982 and 1984 [25]. The three-month mortality rate among current smokers was only one-third of that among ex- and never-smokers combined. In a "final" multivariate model, current smoking had a significant protective effect.

Bettencourt *et al*. [14] and Gaspar *et al*. [16] included consecutive patients with ACS and could not verify the existence of the smoker's paradox. In the latter study the adjusted analysis indicated a higher six-month mortality rate among current and former vs. never-smokers (Figure 1).

In our own study of 381 unselected NSTEMI patients, smokers in the conservative treatment cohort had significantly higher adjusted one-year mortality than non-smokers (including ex-smokers) [7]. Such an increased mortality for smokers was not observed in the invasive cohort (data not published).

#### Registries

A nationwide prospective survey comprised of patients admitted with AMI in all coronary care units (CCU) operating in Israel during a two-month period [17]. Although the six-month mortality rate among smokers was approximately one-third of that among ex- and never-smokers combined, the adjusted analysis could not verify the smoker's paradox.

Within the Hellenic registry of patients admitted to hospital with AMI [11] there was also no evidence of any adjusted in-hospital survival benefit among current vs. non-smokers.

By far the largest registry in this overview was the National Registry of Myocardial Infarction 2 (NRMI 2) [18], with data from 297,458 patients with confirmed AMI admitted to participating hospitals but without hospital transfer. Crude in-hospital mortality among smokers was 50% lower than among the on-average 14 years older non-smokers. A highly significant OR for reduced mortality in the adjusted analysis supported the existence of a "smoker's paradox".

The Análisis del Retraso en el Infarcto Agudo de Miocardio (ARIAM) registry from Spain included patients with AMI and UAP admitted to a CCU/Intensive Care Unit (ICU) [26]. In patients with AMI, the CCU/ICU mortality was nearly one-third among smokers when compared with non-smokers. The adjusted OR for smokers was significantly in favour of the paradox. The Investigación, Búsqueda Específica y Registro de Isquemia Cooronaria Aguda (IBERICA) registry included patients between 25 and 74 years of age admitted to hospital with AMI. Within this registry, smokers had a lower adjusted 28-day mortality rate than the non-smokers used as evidence in favour of the paradox.

The Global Registry of Acute Coronary Events (GRACE) included patients admitted to hospital with a diagnosis of ACS. In an analysis of 19,325 patients the in-hospital mortality rate among smokers was only half of that among never-smokers (3.3% vs. 6.9%). There was no significant difference in adjusted OR for current smokers compared with never-smokers. These results were consistent in all three subgroups of the ACS population studied (STEMI, NSTEMI and unstable angina pectoris).

### Confounding factors included in the adjusted analyses

The confounding variables used in the multivariate analyses (in addition to smoking status) are presented in Table 2. The studies include a wide range of covariates both for baseline risk factors and treatment provided. Four observational studies did not adjust for any treatment provided during hospitalisation [14,16,25,26]. Three registries [15,17,19], in addition to the CADILLAC trial [27], included invasive treatment in the multivariate analyses. The NRMI 2 registry adjusted for "any reperfusion therapy" without specifying the proportion of patients undergoing invasive procedures [18]. Only two studies included renal function in the multivariate analyses [24,25].

**Table 2:** Covariates in addition to smoking status used in the multivariate analyses

Study	Baseline and clinical characteristics	Reperfusion and medication
**Studies supporting the existence of a smoker's paradox**		
Mølstad [25]	Age, atrial fibrillation, s-creatinine, s-potassium	None
Barbash *et al*. [12]	Age, sex, MI site, diabetes, previous MI, antecedent angina, hypertension, hypotension at entry, Killip class, body mass index, hypercholesterolemia, family history of CAD	Time to lysis
GUSTO-1 [13]	Age, sex, systolic blood pressure, Killip class, heart rate, MI site, previous MI, previous CABG, height, diabetes, hypertension, cerebrovascular disease	Time to lysis, type of thrombolytic treatment
NRMI 2 [18]	Age, sex, MI site, previous MI, previous CABG, weight, diabetes, hypertension, hypercholesterolemia, family history of CAD, black race, other race, previous heart failure, previous PTCA, previous stroke, Q vs. non-Q,	Any reperfusion therapy, aspirin first 24 hours, any heparin, intravenous nitroglycerine, beta-blocker, i.v. lidocaine, i.v. magnesium, ACE-inhibitor, calcium channel blocker, other anti-thrombin, other antiplatelet
ARIAM [26]	Age, Killip class, MI site, diabetes, Q-wave, non-Q-wave with ST elevation, non-Q-wave with ST decent	None
IBERICA [15]	Age, sex, MI site, previous MI, diabetes, hypertension, previous angina, spline function for symptoms monitoring, cardiogenic shock or acute pulmonary oedema, severe arrhythmias	Thrombolysis, primary angioplasty, aspirin, beta-blocker
**Studies not supporting the existence of a smoker's paradox**		
Gottlieb *et al*. [17]	Age, sex, systolic blood pressure < 100 mmHg, heart rate > 100/min, Killip class ≥ 2, anterior MI, diabetes, hypertension, previous MI, previous angina, Q-wave MI, family history of CAD, CHF during index hospitalization, atrial fibrillation during hospitalization	Thrombolytic therapy, invasive coronary procedures
GISSI-2 [23]	Age, sex, Killip class, MI site, hypertension, diabetes, previous angina, body mass index, number of leads with ST elevation	Time to lysis
TRACE [21]	Age, sex, body mass index, COPD, previous angina, previous MI, hypertension, family history of CAD, CHF, wall motion index, Q wave anterior MI	Thrombolytic treatment
Andrikopoulos *et al*. [11]	Age, sex, diabetes, hypertension, previous MI	Thrombolytic treatment
OPTIMAAL [20]	Age, sex, COPD, cerebrovascular accidents, diabetes, hypercholesterolemia, hypertension, previous MI, Killip Class, Q wave MI, MI site, peripheral vascular disease	Thrombolytic treatment, discharge medication
Bettencourt *et al*. [14]	Age, sex	None
GRACE [19]	Age, sex, geographical region, previous angina, previous MI, previous PCI/CABG, hypertension, diabetes, hyperlipidemia, chronic heart failure, Killip class, blood pressure, heart rate	Thrombolytic treatment, catheterization, PCI, CABG, aspirin, UFH, LMWH, Glycoprotein IIb/IIIa inhibitor, ACE-inhibitor, calcium channel blocker, beta-blocker, statin
CADILLAC [27]	Age, sex, Killip class ≥ 2, MI site, previous MI, previous CABG, diabetes, hypertension, hypercholesterolemia, LAD culprit vessel, triple vessel disease, baseline TIMI 0 or 3	Stent randomization, abciximab randomization, time from MI to ER, time from ER to first balloon
SYNERGY [24]	Age, gender, creatinine clearance, heart rate, history of CHF, diabetes, baseline rales, ST depression on baseline ECG, weight, peripheral vascular disease, Killip class 3 or 4, No positive biomarkers at randomization, T-wave inversion on baseline ECG	Enoxaparin vs. UFH
Gaspar *et al*. [16]	Age, left ventricular dysfunction, Killip class > 1, ST-elevation ACS	None
Aune *et al*. [7]	Age, s-creatinine, previous left ventricular systolic dysfunction, interaction term (current smoking/strategy)	Invasive strategy, aspirin, statin

ACE-inhibitor, angiotensin converting enzyme inhibitor; CABG, coronary artery bypass grafting; CAD, coronary artery disease; CHF, congestive heart failure; COPD, chronic obstructive pulmonary disease; ER, emergency room; LAD, left anterior descending artery; LMWH, low molecular weight heparin; MI, myocardial infarction; PCI, percutaneous coronary intervention; PTCA, percutaneous transluminal coronary angioplasty; UFH, unfractionated heparin.

## Comments

### Main findings

The smoker's paradox was observed in 6 of the 17 studies included as the basis of this review. One of these studies was an observational single-centre study enrolling unselected AMI patients between 1982 and 1984 [25]. The five other studies dated from the late 1980s and early 1990s and included patients according to the former WHO classification and before the routine use of invasive revascularisation [12,13,15,18,26].

### Possible explanations of the smoker's paradox

The possible explanations for the reported paradoxical findings can be categorised as being either due to systematic errors, residual confounding or different pathogenesis: the latter, therefore, representing a true effect of smoking. Systematic errors would include publication bias. The declining frequency of papers reporting the "smoker's paradox" during the last decade supports our argument that the paradox was the result of skewed reports during the 1980s to 1990s. Another systematic error might be that smokers with an acute cardiac event could have a greater case fatality before admission to hospital than non-smokers [15,30,31]. Those admitted alive to the hospital would, therefore, already represent the survivors. This notion is supported by the fact that the smoker's paradox has only been demonstrated in hospitalised patients.

Adjustment for age and co-morbidity did reduce the magnitude of the smoking effect in many of the studies, but not all. Part of the remaining effect could be due to residual confounding, both because of measurement errors in the co-factors and lack of information about relevant risk factors. The six studies supporting a smoker's paradox have included STEMI patients, with fibrinolysis the dominant reperfusion strategy. This may indicate that there are slight differences in the pathogenesis of the acute coronary event in smokers as compared to non-smokers. It has previously been shown that smokers with STEMI have improved myocardial perfusion after fibrinolysis compared to non-smokers, despite adjustment for differences in age and co-morbidities [32,33]. Tobacco smoking is also associated with increased levels of circulating fibrinogen and tissue factor. This suggests a more fibrin-rich thrombus in smokers with STEMI which would leave them more amenable to fibrinolytic therapy [34] and thus an improved survival rate. All these explanations may operate in unison to contribute to the observation that smokers perform better than non-smoker after an AMI.

### Studies favouring the paradox

#### Randomised trials

The International Tissue Plasminogen Activator/Streptokinase Mortality Trial [12] and GISSI-2 [23] had a similar design and enrolled STEMI patients within the same time period.

A "smoker's paradox" was observed in the International study, whereas only a non-significant trend for better outcome for smokers was demonstrated in GISSI-2. These two studies bring forward the problem of the classification of former smokers. In the International study the OR for six-month mortality was presented for never-smokers vs. current + former smokers, whilst the contrasting GISSI-2 only reported in-hospital mortality in current vs. never-smokers.

In the GUSTO-1 study, 40,599 patients were included in an analysis of 30-day mortality in relation to smoking status. To the best of our knowledge it is in this study that concept of the smokers paradox is first coined. Although not stated expressively in the abstract of the original article, the results from the adjusted analysis were significantly in favour of the paradox in the overall population studied. The abstract refers to the adjusted OR among 2,431 patients subjected to the angiographic substudy, among which the paradox was not apparent.

#### Registries

NRMI 2 reports on 297,458 patients (58%) without hospital transfer out of 510,044 included patients from 1994 to 1997 [18]. The findings are clearly in favour of the paradox. In this report 24% were current smokers compared with 27 to 28% in the overall NRMI 2 population [35]. This indicates that the smokers were more likely to be transferred to other hospitals and hence excluded from the analysis. The rather surprising "paradoxical" protective effects of a family history of CAD, hypercholesterolemia and various medical treatments observed in that model were not commented upon by the authors.

The authors of ARIAM point out that registries in general may have inherent defects due to the possibility of unidentified confounders not included in the analyses [26]. A selection bias may have been present since only patients admitted to the participating hospitals ICU/CCU were included. The IBERICA registry is the only registry supporting the presence of the paradox that incorporated primary PCI in the multivariate regression model. In spite of that, only a minority of patients were subject to such treatment.

The treatment scenario in the late 1980s and early 1990s was quite different from today's practice. Although the preferred treatment for STEMI now is primary PCI, fibrinolysis remains an important alternative to mechanical revascularisation. In Europe, 5 to 85% of patients with STEMI undergo primary PCI [36]. Transfer delays may be unacceptably long before primary PCI is performed, especially for patients living in rural areas or reporting to non-PCI centres. As opposed to the thrombolytic era where the paradox was observed, patients who have had successful thrombolysis should be referred within 24 hours for angiography and revascularization as required [37]. In none of the studies and registries supporting the smoker's paradox was such a treatment strategy applied.

#### The single centre study

The strength of Mølstad's study is the inclusion of consecutive, unselected patients [25]. At that time no reperfusion modalities were available, and the results are purely of historic interest. This study demonstrates the problems related to multivariate analyses of a small patient population, with results being reliant upon the nature and number of the covariates put into the model. When usage of diuretics was added as a fifth covariate in the multivariate model, there was no longer a significant survival benefit for smokers.

### Studies not supporting the paradox

#### Randomised trials

In TRACE some different confounders to those used in the thrombolytic studies were included, with the study recruiting screenees for a randomised trial [21]. The study population that was screened for entry into TRACE is probably representative of unselected AMI patients admitted to hospital alive with an AMI. On the other hand, OPTIMAAL included highly selected patients with AMI and heart failure [20]. The percentage of patients given fibrinolysis was 54% in OPTIMAAL screenees and 39% in TRACE screenees, as opposed to 100% in the fibrinolytic trials. Such differences, along with selection criteria, may explain the different conclusions reached by these studies and the fibrinolytic studies.

In the more recent CADILLAC trial, in which patients were selected to undergo primary PCI for STEMI, the paradox could not be verified [27]. This suggests that the possible existence of a smoker's paradox does not extend into the invasive era.

In SYNERGY, the only randomised trial including NSTE-ACS with patients scheduled for invasive management, a significantly increased adjusted HR for one-year mortality in current vs. never-smokers was found [24].

#### Registries

Both the Israeli [17] and Hellenic [11] registries included hospitalised patients with AMI in the fibrinolytic era. Similar to NRMI 2 [18], IBERICA [15] and ARIAM [26], the mortality rate was compared among current vs. non-smokers, with the results contradictory. It is possible that the number of patients was too small to register the differences noted in the three larger registries.

The GRACE registry was the only study to include patients based upon the current definition of ACS and included in-hospital invasive procedures as a covariate [19]. Neither in the total population of nearly 20,000 patients, nor in the subgroups of patients with STEMI, NSTEMI or UAP, could the existence of the paradox be verified.

#### Single centre studies

In neither of the two single centre studies from Portugal [14,16] could the paradox be demonstrated, with one showing a non-significant increase in odds ratio for current vs. non-smokers for six-month mortality [16] (in keeping with the findings from SYNERGY). In our study of NSTEMI patients a significant interaction between treatment strategy (conservative vs. invasive) and smoking at admission was observed implying a statistically significant effect of smoking on mortality. However, due to the statistically significant interaction term between cohort and smoking, the effect of smoking differed between cohorts. Smokers in the conservative cohort had a statistically significant higher adjusted mortality than non-smokers. In this study smokers received a particular clinical benefit from an early invasive strategy [7], and there was no statistically significant differences between mortality for smokers as compared to non-smokers in the invasive cohort (data not published). Accordingly, there was no evidence for the existence of a smoker's paradox in our study.

## Limitations of the overview

In a systematic search there will always be a conflict between completeness and accuracy. We tried to perform as wide a search as possible and tested the initial search for possible omissions according to known important publications. Nevertheless, we cannot exclude the possibility of having omitted relevant important studies. In that context, two recent studies that did not meet our inclusion criteria are of interest. They address the important smoking interaction of clopidogrel. Desai *et al*. presented data from 3,427 STEMI patients [38]. They found that the beneficial effect of clopidogrel was especially pronounced among those who smoked ≥ 10 cigarettes per day. The other study by Bliden *et al*. of 259 patients undergoing elective stenting shows that clopidogrel induced increased platelet inhibition and lower aggregation as compared with non-smokers [39]. The design of these studies, however, did not allow for the exploration of the existence of the "paradox".

Due to expected variations in the definition of non-fatal cardiovascular events, as well as the sub-classification of fatal events from 1963 onwards, this overview does not explore possible associations between smoking status and events other than total mortality. In addition, the overview does not include any mechanistic studies. Because of the heterogeneity of the data we did not find it meaningful to make a formal meta-analysis.

## Conclusions

The "smoker's paradox" was predominantly observed in AMI patients selected according to the WHO criteria of the 1980s and 1990s. During that time period fibrinolysis was the dominant reperfusion strategy for such patients. The paradox, however, has not been demonstrated in more recent studies using routine early invasive management, although, in one recent study smokers with NSTEMI have been shown to benefit more from an early invasive strategy than non-smokers. As such, we would be wise to encourage smoking cessation rather than relying on the "positive effects" of the so-called paradox.

## Abbreviations

ACE-I: angiotensin converting enzyme inhibitor; ACS: acute coronary syndrome; AMI: acute myocardial infarction; CABG: coronary artery bypass grafting; CAD: coronary artery disease; CCU: coronary care unit; CHF: congestive heart failure; COPD: chronic obstructive pulmonary disease; ER: emergency room; GRACE: global registry of acute coronary events; HR: hazard ratio; ICU: intensive care unit; LAD: left anterior descending artery; LMWH: low molecular weight heparin; MI: myocardial infarction; NSTE-ACS: non-ST-segment elevation acute coronary syndrome; NSTEMI: non-ST-segment elevation myocardial infarction; OR: odds ratio; PCI: percutaneous coronary intervention; PTCA: percutaneous transluminal coronary angioplasty; RCT: randomised controlled trial; RR: relative risk; STEMI: ST-segment elevation myocardial infarction; UAP: unstable angina pectoris; UFH: unfractionated heparin; WHO: world health organization.

## Competing interests

The authors declare that they have no competing interests.

## Authors' contributions

This study was conceived and designed by EA, MM and JEO. The literature search was performed by MM. EA and JEO independently reviewed all titles and abstracts to identify potentially relevant articles. EA, JR, DST and JEO analysed and interpreted the data. EA, DST and JEO drafted the original version of the manuscript. EA, JR, MM, DST and JEO revised the manuscript for critically important intellectual content. All authors have read and approved the final manuscript.

## Pre-publication history

The pre-publication history for this paper can be accessed here:

http://www.biomedcentral.com/1741-7015/9/97/prepub

## Supplementary Material

Additional file 1 **Full search strategy in EMBASE, MEDLINE and Cochrane Central Register of Controlled Trials**. Click here for file
