# Supplementary material for: The "smoker's paradox" in patients with acute coronary syndrome: a systematic review
Source: BMC Med. 2011 Aug 23;9:97. doi: 10.1186/1741-7015-9-97 (PMC3179733; doi:10.1186/1741-7015-9-97)
Supplement: Additional file 1 — Full search strategy in EMBASE, MEDLINE and Cochrane Central Register of Controlled Trials. [file 1741-7015-9-97-S1.PDF]

Database(s): **Ovid EMBASE** 1980 to 2010 Week 37 - Search Strategy:

| # | **Searches** | **Results** |
|---|---|---|
| 1 | Acute coronary syndrome/ | 10523 |
| 2 | Acute heart infarction/ | 33091 |
| 3 | exp Unstable angina pectoris/ | 12634 |
| 4 | *Heart infarction/ | 83138 |
| 5 | (acute adj (coronary syndrome* or ((cardiac or coronary or heart or myocardial) adj (infarct* or event*)) or MI)) or ST-segment elevat* or ST-elevat* or STEMI or NSTEMI or unstable angina*).tw | 74881 |
| 6 | or/1-3,5 | 90393 |
| 7 | Smoking/ or Cigarette smoking/ or Smoking habit/ | 147978 |
| 8 | (nonsmok* or exsmok* or eversmok* or neversmok* or non-smok* or ex-smok* or ((ever or never or former or current) adj smok*) or (smok* adj2 habit*) or (smok* adj3 paradox*) or smok* status*).tw | 51788 |
| 9 | 7 or 8 | 163713 |
| 10 | Mortality/ | 343964 |
| 11 | Death/ or Dying/ or Fatality/ or Heart death/ or Lethality/ or Sudden death/ or "Time of death"/ | 158056 |
| 12 | exp Survival/ | 351229 |
| 13 | Survivor/ | 7203 |
| 14 | Follow up/ | 477390 |
| 15 | (mortal* or death* or endpoint* or end point* or fatality rate* or fatal outcome* or surviv* or dying or die or dies or died).tw. | 1386012 |
| 16 | or/10-15 | 1970568 |
| 17 | letter.pt. | 701045 |
| 18 | editorial.pt. | 354460 |
| 19 | 17 or 18 | 1055505 |
| 20 | 4 and 10 | 9524 |
| 21 | 6 and 16 | 37835 |
| 22 | 20 or 21 | 43342 |
| 23 | 9 and 22 | 2028 |
| 24 | 23 not 19 | 1943 |
| 25 | limit 24 to (danish or english or german or norwegian or swedish) | 1749 |
| 26 | limit 25 to yr="1963 - 1995" | 338 |
| 27 | Acute coronary syndrome/ | 10523 |

| | | |
|---|---|---|
| 28 | Acute heart infarction/ | 33091 |
| 29 | exp Unstable angina pectoris/ | 12634 |
| 30 | ST segment elevation myocardial infarction/ | 1196 |
| 31 | ((acute adj (coronary syndrome* or ((cardiac or coronary or heart or myocardial) adj (infarct* or event*)) or MI)) or ST-segment elevat* or ST-elevat* or STEMI or NSTEMI or unstable angina*).tw | 74881 |
| 32 | or/27-31 | 90455 |
| 33 | *Smoking/ or *Cigarette smoking/ or Smoking habit/ | 59607 |
| 34 | (nonsmok* or exsmok* or eversmok* or neversmok* or non-smok* or ex-smok* or ((ever or never or former or current) adj smok*) or (smok* adj2 habit*) or (smok* adj3 paradox*) or smok* status*).tw | 51788 |
| 35 | 33 or 34 | 90858 |
| 36 | Mortality/ | 343964 |
| 37 | Death/ or Dying/ or Fatality/ or Heart death/ or Lethality/ or Sudden death/ or "Time of death"/ | 158056 |
| 38 | exp Survival/ | 351229 |
| 39 | Survivor/ | 7203 |
| 40 | Follow up/ | 477390 |
| 41 | (mortal* or death* or endpoint* or end point* or fatality rate* or fatal outcome* or surviv* or dying or die or dies or died).tw. | 1386012 |
| 42 | or/36-41 | 1970568 |
| 43 | letter.pt. | 701045 |
| 44 | editorial.pt. | 354460 |
| 45 | 43 or 44 | 1055505 |
| 46 | 32 and 35 and 42 | 617 |
| 47 | 46 not 45 | 601 |
| 48 | limit 47 to (danish or english or german or norwegian or swedish) | 540 |
| 49 | limit 48 to yr="1996 -Current" | 417 |
| 50 | 26 or 49 | 755 |

Database(s): **Ovid MEDLINE(R) In-Process & Other Non-Indexed Citations and Ovid MEDLINE(R)** 1950 to Present

Search Strategy:

| # | Searches | Results |
|---|---|---|
| 1 | Acute Coronary Syndrome/ | 2962 |
| 2 | exp Angina, Unstable/ | 9431 |
| 3 | ((acute adj (coronary syndrome* or ((cardiac or coronary or heart or myocardial) adj (infarct* or event*)) or MI)) or ST-segment elevat* or ST-elevat* or STEMI or NSTEMI or unstable angina*).tw. | 64922 |
| 4 | or/1-3 | 68452 |
| 5 | Smoking/ | 99975 |
| 6 | (nonsmok* or exsmok* or eversmok* or neversmok* or non-smok* or ex-smok* or ((ever or never or former or current) adj smok*) or (smok* adj2 habit*) or (smok* adj3 paradox*) or smok* status*).tw. | 47465 |
| 7 | 5 or 6 | 120709 |
| 8 | mortality/ or "cause of death"/ or fatal outcome/ or hospital mortality/ or survival rate/ | 203200 |
| 9 | death/ or death, sudden/ or death, sudden, cardiac/ | 29082 |
| 10 | Survival/ | 3115 |
| 11 | Survivors/ | 10044 |
| 12 | Follow-Up Studies/ | 412127 |
| 13 | (mortal* or death* or endpoint* or end point* or fatality rate* or fatal outcome* or dying or die or dies or died).tw. | 865633 |
| 14 | or/8-13 | 1314634 |
| 15 | Myocardial infarction/mo | 15151 |
| 16 | letter.pt. | 704863 |
| 17 | comment.pt. | 444085 |
| 18 | editorial.pt. | 272020 |
| 19 | or/16-18 | 1063544 |
| 20 | 4 and 14 | 25619 |
| 21 | 15 or 20 | 33890 |
| 22 | 7 and 21 | 1077 |
| 23 | 22 not 19 | 1057 |
| 24 | limit 23 to (danish or english or german or norwegian or swedish) | 948 |
| 25 | limit 24 to yr="1963 - 1995" | 435 |

| | | |
|---|---|---|
| 26 | Acute Coronary Syndrome/ | 2962 |
| 27 | exp Angina, Unstable/ | 9431 |
| 28 | ((acute adj (coronary syndrome* or ((cardiac or coronary or heart or myocardial) adj (infarct* or event*)) or MI)) or ST-segment elevat* or ST-elevat* or STEMI or NSTEMI or unstable angina*).tw. | 64922 |
| 29 | or/26-28 | 68452 |
| 30 | *Smoking/ | 50643 |
| 31 | (nonsmok* or exsmok* or eversmok* or neversmok* or non-smok* or ex-smok* or ((ever or never or former or current) adj smok*) or (smok* adj2 habit*) or (smok* adj3 paradox*) or smok* status*).tw | 47465 |
| 32 | 30 or 31 | 80927 |
| 33 | mortality/ or "cause of death"/ or fatal outcome/ or hospital mortality/ or survival rate/ | 203200 |
| 34 | death/ or death, sudden/ or death, sudden, cardiac/ | 29082 |
| 35 | Survival/ | 3115 |
| 36 | Survivors/ | 10044 |
| 37 | Follow-Up Studies/ | 412127 |
| 38 | (mortal* or death* or endpoint* or end point* or fatality rate* or fatal outcome* or dying or die or dies or died).tw. | 865633 |
| 39 | or/33-38 | 1314634 |
| 40 | letter.pt. | 704863 |
| 41 | comment.pt. | 444085 |
| 42 | editorial.pt. | 272020 |
| 43 | or/40-42 | 1063544 |
| 44 | 29 and 32 and 39 | 416 |
| 45 | 44 not 43 | 414 |
| 46 | limit 45 to (danish or english or german or norwegian or swedish) | 370 |
| 47 | limit 46 to yr="1996 -Current" | 270 |
| 48 | 25 or 47 | 705 |

**The Cochrane Controlled Trials Register (CENTRAL) (*The Cochrane Library*, Issue 9 2010),**

| ID | Search | Hits |
|---|---|---|
| #1 | MeSH descriptor Acute Coronary Syndrome, this term only | 303 |
| #2 | MeSH descriptor Angina, Unstable explode all trees | 949 |
| #3 | ((acute next (coronary next syndrome* or ((cardiac or coronary or heart or myocardial) next (infarct* or event*)) or MI)) or ST-segment next elevat* or ST-elevat* or STEMI or NSTEMI or unstable next angina*):ti,ab | 7532 |
| #4 | (#1 OR #2 OR #3) | 7740 |
| #5 | MeSH descriptor Smoking explode all trees | 4477 |
| #6 | (nonsmok* or exsmok* or eversmok* or neversmok* or non-smok* or ex-smok* or ((ever or never or former or current) next smok*) or (smok* near/2 habit*) or (smok* near/3 paradox*) or smok* status*):ti,ab | 3974 |
| #7 | (#5 OR #6) | 7114 |
| #8 | MeSH descriptor Mortality, this term only | 359 |
| #9 | MeSH descriptor Cause of Death, this term only | 935 |
| #10 | MeSH descriptor Fatal Outcome, this term only | 17 |
| #11 | MeSH descriptor Hospital Mortality, this term only | 866 |
| #12 | MeSH descriptor Survival Rate, this term only | 6816 |
| #13 | MeSH descriptor Death, this term only | 52 |
| #14 | MeSH descriptor Death, Sudden, this term only | 143 |
| #15 | MeSH descriptor Death, Sudden, Cardiac, this term only | 433 |
| #16 | MeSH descriptor Survival, this term only | 93 |
| #17 | MeSH descriptor Survivors, this term only | 419 |
| #18 | MeSH descriptor Follow-Up Studies, this term only | 34481 |
| #19 | (mortal* or death* or endpoint* or end point* or fatality rate* or fatal outcome* or dying or die or dies or died):ti,ab | 46716 |
| #20 | (#8 OR #9 OR #10 OR #11 OR #12 OR #13 OR #14 OR #15 OR #16 OR #17 OR #18 OR #19) | 78914 |
| #21 | (#4 AND #7 AND #20) | 85 |
